# Safe practice and nursing care of patients receiving oral anti-cancer medicines: a position statement from UKONS

**DOI:** 10.3332/ecancer.2010.177

**Published:** 2010-01-26

**Authors:** C Oakley, E Lennan, H Roe, O Craven, K Harrold, C Vidall

**Affiliations:** 1 Guy’s and St Thomas’ NHS Foundation Trust, London, UK; 2 Southampton University Teaching Hospital Trust, Southampton, UK; 3 North Cumbria University Hospitals NHS Trust, Cumbria, UK; 4 Christie Hospital, Manchester, UK; 5 Mount Vernon Cancer Centre, Northwood, UK; 6 Healthcare at Home, Staffordshire, UK

## Abstract

This position statement has been formulated by oncology nurses from the UK Oncology Nursing Society (UKONS) to provide guidance for nurses on safe practice with oral anti-cancer medicines. The ultimate aim is to ensure that patients are not only well informed and capable of managing their oral anti-cancer medicines, but also supported safely and effectively while they are receiving these treatments.

## Background

The past decade has seen a change in the way many patients with cancer are managed. There has been a huge increase in the number of oral anti-cancer medicines licensed for the treatment of solid and haematological tumours, and this has led to a rapid escalation of their use. During 2006–7, for example, nearly 24 million doses of oral anti-cancer medicines were used in England alone [1].

The enthusiastic uptake of oral anti-cancer medicines has been driven by the advantages they offer to patients and to the NHS, in terms of toxicity management, convenience of delivery and cost-effectiveness. Compared with intravenous (IV) cancer treatments, oral drugs require fewer NHS resources. Patients may not need admission to the ward or day unit for cannulation and administration of drugs (unless receiving IV anti-cancer medicines in combination with the oral drug). This helps to reduce the burden on inpatient beds and on nursing and pharmacy staff in already stretched chemotherapy units. Furthermore, patients reportedly prefer oral to IV anti-cancer medication because of the convenience of self-administering treatment at home [2].

However, while chemotherapy services recognize standards advocating the administration of IV cancer treatments by appropriately trained nursing staff (as outlined in the ***Manual for Cancer Services*** [3]), a recent survey of UK pharmacists suggests that procedures for the administration of oral anti-cancer medicines are less rigorous [4]. This has worrying consequences: in January 2008, the National Patient Safety Agency (NPSA) issued a rapid response report on the potential for fatal outcomes if incorrect doses of oral anti-cancer medicines are administered [1,5]. This was in response to three deaths and 400 patient safety issues involving oral anti-cancer medicines reported between November 2003 and July 2007. According to the NPSA, half of the reports concerned the wrong dosage, frequency, quantity or duration of oral anti-cancer treatment. Of further concern are that patient errors may be under-reported [6], and the finding of the National Confidential Enquiry into Patient Outcome and Death (NCEPOD) that only 35% of patients who died within 30 days of receiving systemic anti-cancer medicines (oral or IV) were judged to have received ‘good’ care [7]. In light of these reports, there is clearly a need for improved systems covering the management of patients receiving oral therapies.

Without a structured approach to delivery of oral anti-cancer treatments, we cannot be sure patients are regularly and adequately assessed and reviewed by appropriately qualified healthcare professionals. This may leave patients and carers feeling overwhelmed by the responsibility of managing their own treatments [2,6]. Patient errors may consequently include incorrect dosing or late reporting of treatment-related toxicities.

The National Chemotherapy Advisory Group (NCAG 2009) [8] states that the same governance frameworks and care processes used for IV chemotherapy should equally apply to oral chemotherapy. The British Oncology Pharmacy Association has already published a position statement with the aim of improving preparation and dispensing of oral anti-cancer medicines [9]. UKONS believes that it is now time to provide guidance for nurses. The role of this position statement is therefore to outline the specialist knowledge and skills required to ensure the safe and competent delivery of oral anti-cancer medicines and the appropriate care and monitoring of patients receiving these treatments.

## Definitions

The commonly understood definition of oral anti-cancer medicines covers all drugs with direct anti-tumour activity, orally administered to cancer patients [9]. The definition includes traditional cytotoxic drugs as well as targeted therapies (see Table 1), but does not include hormonal or anti-hormonal agents, such as tamoxifen or anastrozole. Drugs fitting this definition are included in the remit of this document.

## Principles of safe practice

In general, the principles of safe practice for administration of oral anti-cancer medicines should be the same as those for IV treatments, as outlined in the ***Manual for Cancer Services*** [3]. Since patients are required to manage their own treatments away from hospital, additional safe practice procedures are needed to ensure that patient education is optimal and patients are competent in managing their treatment.

### Standards, protocols and guidelines

Guidelines and protocols are required for oral anti-cancer medications [1,3], covering the supply of treatment (e.g. procedures for ensuring patients are adequately prepared and supported to manage oral therapies, and verifying the patient’s identity before treatment is dispensed). Providers should also ensure those managing patients on oral anti-cancer medications have the appropriate specialist skills and knowledge to ensure safe administration [3].

### Training

Within a clinical chemotherapy service, registered nurses who administer anti-cancer medicines should have undertaken degree-level cancer-related studies, including one or more modules in chemotherapy, and their competence should be assessed annually [3]. In relation to oral treatment, the training programme should include the following:

- knowledge of oral anti-cancer medication protocols, as detailed in the network list of approved regimens [3];
- cancer drug administration;
- establishing and running nurse-led clinics;
- communication skills;
- assessment of patients receiving anti-cancer medication, which is incorporated into an ongoing holistic assessment;
- assessment of common anti-cancer medication side effects, including myelosuppression, nausea, vomiting, stomatitis, diarrhoea, hypertension, lethargy and palmar-plantar erythrodysesthaesia;
- cancer treatment side effects that constitute a medical emergency, for example neutropenic sepsis, thrombosis and angina;
- health and safety aspects of anti-cancer medication administration, including storage, safe handling and waste disposal.

## Prescribing

The prescribing policy for oral anti-cancer medicines should be the same as for IV anti-cancer medication, as outlined in the ***Manual for Cancer Services*** [3]. All oral anti-cancer medicines should be prescribed only in the context of a written protocol and treatment plan [1].

### Nurse prescribing and treatment initiation

In line with current health policy, nursing and pharmacy roles are being expanded to take on responsibilities that were traditionally the role of doctors, such as prescribing. Nurse prescribing in oncology enables the patients to get the required medication on time and may allow patients to be managed nearer to home [10]. It facilitates a holistic approach to patient care, and it enables sharing of the prescribing workload in busy chemotherapy clinics [7,11].

The ***Manual for Cancer Services*** [3] states that oral anti-cancer treatment should be initiated by a cancer specialist. These standards have been adapted in certain networks to allow non-medical prescribers to prescribe the first cycle (e.g. The Christie NHS Foundation Trust and Southampton University Hospitals Trust). Variations in local policy must be acknowledged and underpinned by appropriate continuous professional development and robust governance arrangements.

Regardless of whether the prescriber is medical or non-medical, it is important that he or she has access to and follows local written protocols, including guidance on monitoring and treatment of toxicity [1,7].

### Prescriptions

Where possible, electronic prescribing systems should be used because these have been shown to help standardize protocol-based prescribing and reduce the risk of errors [8]. If an approved and validated electronic prescribing system is unavailable [4], all prescriptions of anti-cancer medicines should be computer generated, at least when using regimens from the agreed list [3]. All of these prescriptions should be checked and authorized by an appropriately trained pharmacist [3].

### Prescription verification procedures

Nurses are well placed to check patient suitability for oral anti-cancer medicines, for example by noting whether cognitive state, social isolation or presence of severe dysphagia prevents administration of anti-cancer medication by this route [7]. It is important that label directions are clear and unambiguous and that they include, where relevant, the intended period of treatment, start and stop dates, any special instructions and appropriate information about the need for safe handling [9].

When patients are self-administering oral anti-cancer medicines in their own home, it is important that they [1]:

- are aware of the required monitoring arrangements;
- have access to information in the written protocol and treatment plan from the hospital where treatment was initiated;
- have access to advice from an appropriately qualified healthcare professional (according to training requirements outlined on page 6 of the ***Manual for Cancer Services*** [3]) with experience in cancer treatment in that hospital;
- are prescribed and dispensed only sufficient medication for that cycle (i.e. until their next scheduled assessment).

## Patient education and information

Equipping patients and carers to manage oral anti-cancer medications is vital to patient safety, patient concordance and treatment success. Cancer nurses and/or pharmacists with specialist anti-cancer medication knowledge can ensure that patients receive comprehensive education fit for their needs [9].

It is recommended that patient preparation takes place within dedicated specialist clinics. All cancer services should therefore urgently assess the potential role of nurse-led/pharmacist-led clinics in the management of patients prescribed oral anti-cancer therapies [8,12].

### Pre-treatment consultation

Unique to oral anti-cancer medicines is the need to educate patients on self-administration of anti-cancer medication, including the principles of safe handling, storage and disposal, according to the chemotherapy services and Control of Substances Hazardous to Health Regulations 2002 (as amended) [9,13]. The pre-treatment consultation should be carried out by an appropriately trained healthcare professional (according to training requirements outlined on page 6 of the ***Manual for Cancer Services*** [3]) to ensure that patients fully understand how and when to take their oral anti-cancer medicines [9].

The pre-treatment consultation should be structured and supported by nursing protocols and checklists, such as the Multinational Association for Supportive Care in Cancer Oral Agent Teaching Tool [14]. The consultation should be tailored to the individual patient’s needs, knowledge, perception and psychological status to enable them to self-manage their treatment. Understanding should be checked before the patient is given their oral medication [9]. It is further recommended that expected patient response to serious side effects, such as neutropenic sepsis, are rehearsed with patients and carers, and that key points are reviewed at the end of the consultation to ensure optimal management of complications.

The hospital should provide patients with comprehensive, evidence-based written, verbal and pictorial advice giving clear, unambiguous instructions about their oral anti-cancer medications. Full use should be made of NHS cancer websites to provide information for patients and carers to ensure the safe use of oral anti-cancer medicines [1]. Patient alert cards [15] and scheduling diaries [6] are also useful when assisting patients to manage their own treatment. Patients should be encouraged to record side effects when they occur and have this record with them when they are next assessed.

Topics for discussion (supported by written information) include the following [3,9]:

- the intended oral anti-cancer regimen and treatment plan including, doses, route of administration, cycles intended, investigations, monitoring procedures, management of toxicities and any required supportive care measures or medications; [1]
- local pathway for treatment and management of complications;
- when and how to take the tablets correctly (e.g. with or before food);
- what to do in the event of missed doses;
- what to do if patients vomit after taking a dose;
- what side effects to expect and what to do about them,
- which symptoms can patients manage at home?
- at what level of severity should they contact the hospital?
- how side effects will be managed;
- potential consequences of not presenting early with symptoms;
- why and how to obtain further supplies;
- what role their GP/community nurses are expected to play in their treatment;
- principles of safe handling, storage and disposal of medications and delivery devices (e.g. plastic spoons), and any implications for family members;.
- possible interactions with other drugs, supplements or herbal remedies.

Key information points should be reinforced and holistic patient needs explored on subsequent visits [9].

## Consent

Chemotherapy nurses should be actively involved in the patient consent process and may legally take written consent from patients prescribed oral therapies if supported by local Trust policy. The British Committee for Standards in Haematology guidelines [16] have been endorsed by NCEPOD [7] to assist in the reduction in mortality rates within 30 days of systemic anti-cancer chemotherapy; they note that [17] ‘The healthcare professional carrying out the procedure is ultimately responsible for ensuring the patient is giving valid consent, and it is they who will be held responsible in law if this is challenged later. However, team-work is now the way the National Health Service operates and, where written consent is being sought, it may be appropriate for several members of the team to participate in the process of seeking consent. It is the responsibility of the healthcare professional to ensure that when he/she requires a colleague to seek consent on his/her behalf he/she is confident that the colleague will work within his/her own competence and will not agree to perform tasks which exceed that competence’. NCEPOD [7] requires the consent process to include a clear outline of possible side effects, including those that are potentially life threatening. Consequently, standardized consent forms will be developed through a national work stream.

### Access to 24-hour specialist oncology advice

Clinical chemotherapy services should provide a 24-hour telephone advice service for patients prescribed oral therapies, with appropriately trained nursing, medical or pharmacy staff handling queries [3]. Patients can use such services to seek advice on anti-cancer medication management including side effects, and how to obtain help and treatment for them. It is important that this information is well presented and consistent with other information that the patients are given by the healthcare team [7].

NCAG recommends that chemotherapy telephone advice lines should be supported by dedicated acute oncology services (AOS) [8]. These new services will encompass the management of patients who develop severe complications following chemotherapy, including neutropenic sepsis. Chemotherapy-trained nurses have essential skills to support AOS’s and should actively engage/lead in their development.

## Monitoring and follow-up

Monitoring of patients who have been prescribed oral anti-cancer medicines is important for safe care because it helps to identify toxicities and complications early, allows prompt remedial actions and prevents serious complications and possible hospitalization [7,8,12]. Monitoring of patients may be particularly important for newer oral anti-cancer therapies, with novel modes of action as these may have unexpected side effects [17]. Nurses should strongly consider establishing proactive targeted support services to identify problems before they become serious (e.g. using routine telephone monitoring) [8,12].

### On-treatment assessment

All patients should be seen by a healthcare professional with specialist knowledge before every treatment cycle [9]. For each patient, records should be made of the following [3]:

- continued ability to manage treatment at home, patient and carer needs and referral to other services;
- performance status;
- toxicity assessment, including grade, duration, management and relevance and any change in supportive medications;
- the results of essential serial investigations applicable to that cycle (and before administration within a cycle, if applicable);
- any dose modifications, and whether they are intended to be permanent;
- any cycle (or administration) delays.

After the final cycle of a treatment course, the records for each patient should include the following [3]:

- whether the course was completed or not;
- if the course was not completed or if the planned dose was reduced, the reasons for cessation or reduction;
- for completed courses of non-adjuvant treatment, a reference to the response;
- the plans for ongoing review and support.

### The role of the primary care team

The expanding availability of oral anti-cancer medicines means that communication with primary care and community health practitioners is increasingly important. Primary care nurses and GPs are among those who should be given precise and regular information on a patient’s treatment, potential side effects and drug interactions, where to get advice, where patients should report to and how they should be managed in the event of treatment complications, such as the following [3,7]:

- neutropenic sepsis;
- nausea and vomiting;
- stomatitis, other mucositis;
- palmar-plantar erythrodysesthaesia;
- diarrhoea;
- hypertension;
- angina.

There are increasing opportunities for shared care between secondary and primary care providers [8]. While treatment is initiated by specialists, follow-up and monitoring may be shared. Where shared care is in place, protocols and guidelines should be developed with primary care providers.

### Clinical governance

Cancer service providers should develop robust quality improvement systems, to ensure that protocols and policies detailed in national guidance are developed, implemented and regularly audited [7,8]. Each chemotherapy service should have regular morbidity/mortality meetings to review practice and policies and procedures in relation to the safety and quality of chemotherapy [7]. Audit should include referral processes, for example management of treatment-related emergencies and key outcomes of treatment [3]. In addition, the cancer service provider should regularly review incidents and errors, and subsequent reports should be reviewed by the network chemotherapy group and local governance forums [8].

This position statement will be reviewed by oncology nurses from the UK Oncology Nursing Society in 2012, or sooner if warranted.

## Figures and Tables

**Table 1:**
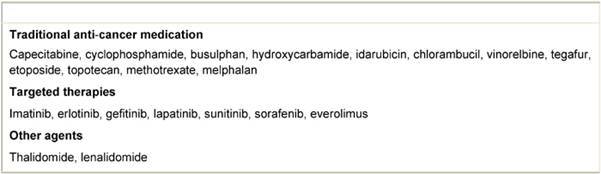
Examples of oral anti-cancer medicines [18]
